# Norovirus Translation Requires an Interaction between the C Terminus of the Genome-linked Viral Protein VPg and Eukaryotic Translation Initiation Factor 4G*

**DOI:** 10.1074/jbc.M114.550657

**Published:** 2014-06-13

**Authors:** Liliane Chung, Dalan Bailey, Eoin N. Leen, Edward P. Emmott, Yasmin Chaudhry, Lisa O. Roberts, Stephen Curry, Nicolas Locker, Ian G. Goodfellow

**Affiliations:** From the ‡ Section of Virology, Department of Medicine, Imperial College London, Norfolk Place, London W2 1PG, United Kingdom,; § School of Immunity and Infection, College of Medical and Dental Sciences, University of Birmingham, Birmingham B15 2TT, United Kingdom,; ¶ Department of Life Sciences, Imperial College London, South Kensington, London SW7 2AZ, United Kingdom,; ‖ Division of Virology, Department of Pathology, University of Cambridge, Addenbrooke's Hospital, Hills Road, Cambridge CB2 2QQ, United Kingdom, and; ** University of Surrey, Faculty of Health and Medical Sciences, School of Biosciences and Medicine, Guildford GU2 7HX, United Kingdom

**Keywords:** Eukaryotic Translation Initiation Factor 4E (eIF4E), Plus-stranded RNA Virus, Translation, Translation Initiation Factor, Virus, VPg, eIF4G, Norovirus

## Abstract

Viruses have evolved a variety of mechanisms to usurp the host cell translation machinery to enable translation of the viral genome in the presence of high levels of cellular mRNAs. Noroviruses, a major cause of gastroenteritis in man, have evolved a mechanism that relies on the interaction of translation initiation factors with the virus-encoded VPg protein covalently linked to the 5′ end of the viral RNA. To further characterize this novel mechanism of translation initiation, we have used proteomics to identify the components of the norovirus translation initiation factor complex. This approach revealed that VPg binds directly to the eIF4F complex, with a high affinity interaction occurring between VPg and eIF4G. Mutational analyses indicated that the C-terminal region of VPg is important for the VPg-eIF4G interaction; viruses with mutations that alter or disrupt this interaction are debilitated or non-viable. Our results shed new light on the unusual mechanisms of protein-directed translation initiation.

## Teaser

**Background:** Noroviruses use a virus-encoded protein, VPg, covalently linked to the viral RNA for translation.

**Results:** The direct interaction of VPg with the central domain of eIF4G is required for norovirus translation.

**Conclusion:** eIF4G plays a central role in norovirus VPg-dependent translation initiation.

**Significance:** The VPg-eIF4G interaction may provide a suitable target for the specific inhibition of norovirus translation.

## Introduction

As obligate intracellular pathogens, viruses rely exclusively on the host cell for the components of the protein synthesis machinery and have evolved a multitude of mechanisms to enable the translation of viral mRNA into protein in the presence of high concentrations of competing cellular mRNA (1, 2). The translation of mRNA into protein is the final stage in the process of gene expression. The key regulatory step in this process is the initiation phase, a multistep process that results in recruitment of the ribosomal subunits to the start codon on the mRNA. Initiation requires the participation of a number of eukaryotic initiation factors (eIFs)^3^ to assemble a 43 S preinitiation complex on an mRNA molecule before completion of the 80 S initiation complex at the initiation codon. Cytoplasmic mRNAs are capped at the 5′ end, and the first step in translation is the binding of the initiation factor eIF4E, a component of the eIF4F complex, to the 5′ cap structure. eIF4F is a complex of three initiation factors; eIF4E is the cap-binding protein, eIF4A functions as an RNA helicase, and eIF4G acts as a scaffold to bridge the mRNA to the 40 S ribosomal subunit via its interaction with eIF3 (3). eIF4G contains three “domains”; the N-terminal one-third contains the eIF4E-binding site (4, 5) and a binding site for poly(A)-binding protein (6), the middle third contains binding sites for eIF3 and eIF4A (7), and the C-terminal third contains another eIF4A-binding site (7) as well as a binding site for the regulatory kinase, Mnk-1. After the 43 S ribosomal complex is bound to the 5′-cap of the mRNA, it scans in the 5′ to 3′ direction to find the AUG initiation codon, at which point the 60 S ribosomal subunit joins, forming an 80 S initiation complex that commences protein synthesis.

Noroviruses, members of the *Caliciviridae* family of small positive-strand RNA viruses, are a major cause of acute gastroenteritis in man (8) but have also been identified in a number of other species including dogs (9, 10), cats (11), sheep (12), and cattle (13). Recent data indicate that noroviruses may also be associated with more significant clinical diseases including necrotizing enterocolitis and benign seizures in infants (14–17). Despite the significant economic impact of noroviruses and numerous ongoing efforts, human noroviruses have yet to be cultivated in the laboratory (18). Our understanding of norovirus biology has been greatly enhanced by the discovery of murine norovirus (MNV) in 2003 (19), which remains the only norovirus that can be cultivated in the laboratory (20). Combined with the availability of various reverse genetics systems (21–23), MNV provides a valuable system with which to dissect the norovirus life cycle and has greatly increased our understanding of the molecular mechanisms of norovirus genome translation and replication (for review, see Ref. 24).

We previously demonstrated that the feline calicivirus (FCV), MNV, and human norovirus VPg proteins can interact directly with the cap-binding protein eIF4E (25, 26). A similar interaction of the plant potyvirus VPg with eIF4E has also been reported and is known to determine the relative susceptibility of plant species to infection (for review, see Ref. 27). Functional analyses of these interactions in calicivirus VPg-dependent translation have indicated that eIF4E is essential for FCV translation as depletion of eIF4E or inhibition of the eIF4E-eIF4G interaction ablates FCV translation. Importantly, however, despite the interaction between the norovirus VPg protein and eIF4E, it appears to be dispensable for MNV translation initiation, at least *in vitro* (25). Therefore to date, no functional role for a VPg-eIF4E interaction has been described. FCV and MNV belong to the *Vesivirus* and *Norovirus* genera of the *Caliciviridae*; therefore, although a significant degree of similarity is likely to exist in their mechanism of genome translation and replication, subtle differences in the relative requirements for cellular factors between different genera is likely. Previous reports have also highlighted a potential association of the norovirus VPg protein with components of the eIF3 complex (28) and eIF4E, PABP, and eIF4G as well as the ribosomal protein S6 (29), although the functional relevance of these interactions has yet to be demonstrated. Here we describe the proteomic characterization of the murine norovirus translation initiation factor complex, demonstrating that VPg associates directly with the core components of the eIF4F complex and PABP. We further demonstrate that the interaction between eIF4G and VPg is essential for norovirus translation. Furthermore, we demonstrate that eIF4G is required for efficient virus replication in cell culture.

## EXPERIMENTAL PROCEDURES

### 

#### 

##### Cells, Plasmids, and Antibodies

Murine macrophage RAW264.7 and microglial BV2 cell lines were cultured in Dulbecco's modified Eagle's medium (DMEM) with 10% (v/v) fetal calf serum (FCS), penicillin (100 units/ml), streptomycin (100 μg/ml), and 10 mm HEPES buffer. Baby hamster kidney cells (BHK-21) expressing T7 RNA polymerase (BSRT7 cells) were cultured in similar media lacking HEPES but containing 1 mg/ml G418. Similarly, HEK 293T cells were maintained in media lacking HEPES. HEK 293T cells stably expressing pMEP4-NTAP or pMEP4-NTAP MNV VPg plasmids were supplemented with 50 μg/ml hygromycin B and nonessential amino acids. The HEK293 TREX cells stably expressing pcDNA4/TO-NTAP derivatives of MNV VPg were supplemented with 5 μg/ml Blasticidin and 200 μg/ml Zeocin. All cell lines were maintained at 37 °C and 10% CO_2_.

The constructs pET28a:HIS N-FAG_1–532_, pET28a:HIS C-FAG_1177–1600_, pET28a:HIS M-FAG_533–1176_, pET28a:HIS p100_675–1600_, and pET28a:HIS 4GM_654–1131_, expressing the truncated eIF4GI proteins N-FAG, C-FAG, M-FAG, p100, and 4GM, respectively, were kindly provided by Simon Morley (University of Sussex). Infectious clones of wild type MNV (pT7:MNV 3′RZ) or an MNV mutant with an alanine substitution at position Phe-123 of the VPg protein, F123A (pT7:MNV 3′RZ F123A), were used in various experiments. pCDNA4/TO-FLAG 4GM was generated by PCR amplification of 4GM from pET28a:HIS 4GM using forward (GTAAAGCTTGCCACCATGGATTACAAGGATGACGACGATAAG*GATCC*CACTAGACTAC) and reverse (TGGCTCGAGTCATACCGCTTGTTGAAG) primers to generate a HindIII/XhoI fragment, which was inserted into pCDNA4/TO.

Antisera to the various MNV non-structural proteins were generated in-house and have been described previously (22, 25). Other antisera was sourced as follows: eIF4E (Santa Cruz; sc-13963), eIF4A (Santa Cruz; sc-14211), PABP (New England Biolabs; 4992), eIF4G (2498; New England Biolabs), GAPDH (AM4300; Ambion), and the histidine tag (HIS; Santa Cruz).

##### Tandem Affinity Purification

A detailed overview of this protocol is available elsewhere (30). Two expression systems, tetracycline inducible system (pCDNA6/TR and pcDNA4:TO NTAP) and cadmium chloride (CdCl_2_) inducible system (pMEP4-NTAP), were used for expressing genes of interested, *i.e.* genes encoding MNV VPg. Both inducible systems expressed a GS tag (containing two protein G units and streptavidin binding peptide). For the CdCl_2_ inducible system, the transfected 293 cells were selected with 50 μg/ml hygromycin B (Roche Applied Science) and were later induced with 10 μm CdCl_2_ for 16 h. For the tetracycline inducible system, 293 T-REx cells were selected with 5 μg/ml Blasticidin and 200 μg/ml Zeocin after a 24-h post-transfection of plasmid. A single colony was isolated from the cell population to examine protein expression. ∼2 × 10^8^ cells expressing a protein of interest were induced with 1 μg/ml doxycycline for 16 h. Tandem affinity purification (TAP) pulldown assays were then performed on lysates prepared from these cells as described previously (30). Eluted proteins were concentrated using VivaSpin 500^TM^ column (Vivascience). Samples were subject to SDS-PAGE gel electrophoresis and stained with Coomassie Blue. The whole gel lane was divided into either 25 or 9 parts as appropriate, excised, and analyzed by mass spectrometry (Genome Quebec Innovative Center, McGill University).

The McGill University and Genome Quebec Innovation Centre, Canada, performed all mass spectrometry (MS). Proteins present in bands excised from SDS-PAGE gels were identified using a quadrupole TOF micro-mass spectrometer (Waters Micromass) as described previously (31). In this case, however, Mascot was set up to search a *Homo sapiens* database. Scaffold (Proteome Software Inc.) was used to validate tandem MS (MS/MS)-based peptide and protein identifications. Similar purifications and mass spectrometry analyses were performed with cell lines expressing the GS tag alone to provide a list of nonspecific interactions, which were then removed from the data set. Those remaining were considered VPg-specific interacting proteins.

##### m7GTP-Sepharose Chromatography

BSRT7 cells were used to overexpress viral proteins from full-length cDNA constructs as described below for the reverse genetics recovery of MNV. At 18 h post-transfection cells were lysed in 120 μl of CAP lysis buffer (1% Triton X-100, 100 mm KCl, 0.1 mm EDTA, 10% glycerol, 2 mm MgCl_2_, 20 mm Hepes. pH 7.6, and protease inhibitors mixture) and were incubated on ice for 5 min at 4 °C. Non-lysed cells and debris were removed by centrifugation at 15,000 × *g* for 5 min. The cleared lysates were subsequently incubated with prewashed m7GTP-Sepharose (25 μl bead volume; GE Healthcare) along with an additional 120 μl of CAP lysis buffer (without 1% Triton X-100) for 2 h at 4 °C to allow binding to take place. Any unbound proteins were later removed by centrifugation at 1000 × *g* for 5 min followed by washing 3 times using lysis buffer without Triton X-100. Finally, proteins associated with the cap structure were eluted with SDS-PAGE reducing sample buffer and analyzed by Western blot after subjected to SDS-PAGE electrophoresis.

##### RNA Affinity Chromatography

Ribosome-bound MNV RNA translation complexes were purified as described previously (32). Briefly, 2.5 μg of purified VPg-linked MNV RNA or *in vitro* transcribed HCV IRES (as described in Ref. 33) were pre-annealed to biotinylated DNA oligonucleotides targeting the 3′ end of the MNV genome (5′-GCATCTAACTACCACAAAGAAAAGAAAGC-3′; 5′-GCCCTGCTACTCCCGATCTTAGGG-3′) or the HCV IRES construct (5′-GGGATTTCTGATCTCGGCG-3′; 5′-TTTCTGATCTCGGCGTCTA-3′) and translated for 15 min at 30 °C in rabbit reticulocyte lysates pretreated with 5 mm GMP-PNP and 0.5 mm puromycin before the addition of 100 μg/ml cycloheximide. Translation complexes were subsequently immobilized onto Streptavidin Dynabeads M-280 (Invitrogen). After extensive washing in THEMK buffer (34 mm Tris, 66 mm HEPES, 0.1 mm EDTA, 2.5 mm MgCl_2_, 75 mm KCl, pH 7.8), the complexes assembled onto MNV RNA or HCV IRES were released by adding RNase H. The presence of individual eIFs was then assayed by immunoblotting as described above and compared with a control lane containing rabbit reticulocyte lysate.

##### Reverse Genetics Recovery of Mutant Viruses

Reverse genetics recovery of recombinant MNV was performed by infecting baby hamster kidney cells with fowlpox virus expressing T7 RNA polymerase followed by transfection of MNV cDNA expression constructs as described previously (22, 34). To analyze protein expression, cells were harvested 24 h post-transfection for Western blot analysis. Virus yield was determined by TCID50.

##### Identification of the Minimal VPg Binding Domain within eIF4GI

Constructs expressing the truncated eIF4G proteins, N-FAG, C-FAG, M-FAG, p100, and 4GM (described above) were co-transfected with either an infectious clone of wild type MNV or MNV containing an alanine substitution at position Phe-123 of the VPg protein (F123A) into FPV-infected BSRT7 cells using Lipofectamine 2000 (Invitrogen). pcDNA3.1+ was used as a negative control. At 24 h post-transfection, the cells were lysed in 120 μl of CAP lysis buffer (1% Triton X-100 mm KCl, 0.1 mm EDTA, 10% glycerol, 2 mm MgCl_2_, 20 mm Hepes, pH 7.6, and protease inhibitor mixture) and centrifuged at 13,000 × *g* in a benchtop microcentrifuge to pellet insoluble debris.

The cleared lysate was then added to prewashed Ni-CAM beads, and an additional 120 μl of CAP lysis buffer lacking Triton X-100 was added. The reaction mix was incubated on a rotary mixer at 4 °C overnight. The beads were then washed 5 times using a more stringent CAP buffer (200 mm KCl, 0.1 mm EDTA, 10% glycerol, 2 mm MgCl_2_, 20 mm Hepes, pH 7.6) in the absence of Triton X-100. Proteins were eluted by resuspending the beads in 45 μl of SDS sample buffer and boiling the samples for 5 min. Purified protein was resolved in an SDS-PAGE gel and analyzed by Western blotting.

##### His-VPg:eIF4G Pulldown Assay

The pETM11 plasmid encoding MNV VPg 1–124 is as described elsewhere (35). QuikChange site-directed mutagenesis (Stratagene) was used to produce the MNV VPg 1–124 F123A mutant. eIF4GI 4GM (652–1132) was amplified by PCR from a human eIF4GI clone (NCBI accession number AAM69365.1) and cloned into a modified pGEX2T vector using BamHI and EcoRI restriction enzymes (primer details available upon request). GST and GST-eIF4GI 4GM were expressed in *Escherichia coli* (DE3) Rosetta and *E. coli* (DE3) CodonPlus cells, respectively. Expression was induced during mid-log phase by the addition of 1 mm final isopropyl 1-thio-β-d-galactopyranoside in the case of GST for 4 h at 37 °C and for 3.5 h at 30 °C for GST-eIF4GI 4GM. Wild type and F123A MNV VPg were expressed as described previously (35).

GST and GST-eIF4GI 4GM were purified by affinity chromatography using Glutathione-Sepharose 4B resin (GE Healthcare). GST was dialyzed against 50 mm Tris buffer, pH 7.6, 200 mm NaCl, and 2 mm β-mercaptoethanol. GST-eIF4GI 4GM was dialyzed against the same buffer but with 150 mm NaCl and then further purified by size exclusion chromatography using a Superdex S200 10/300 column (GE healthcare). Both proteins were finally dialyzed against 50 mm Tris buffer, pH 7.6, containing 150 mm NaCl (binding buffer). His-tagged MNV VPg proteins (wild type and the F123A mutant) were purified by affinity chromatography to TALON resin (Clontech) and subsequently dialyzed against 50 mm sodium phosphate, pH 6.5, 300 mm NaCl, and 1 mm DTT.

In pulldown experiments 25 μl of TALON beads were used to capture the bait protein (His_6_-tagged MNV VPg WT or F123A at a final concentration of 5.5 μm) and GST or GST-eIF4GI were used as prey proteins as a final concentration of 1.1 μm. The proteins were mixed together with 450 μl of binding buffer in SigmaPrep spin columns (Sigma) and incubated by rotation at 4 °C for 90 min before collection of the flow through fraction. The resin was washed with 750 μl of binding buffer then twice with 10 mm imidazole in 750 μl of binding buffer. The bait was eluted with 100 μl of 500 mm imidazole in binding buffer. Captured proteins were analyzed by SDS-PAGE.

##### siRNA Knockdown and Reconstitution Experiments

eIF4GI-siRNA-mediated reduction of protein expression was performed in HEK 293T cells. Cells were transfected with either control nonspecific siRNA (GCGCGCUUUGUAGGAUUCG) or eIF4GI-specific siRNAs (CCCAUACUGGAAGUAGAAG). At 24 h post-transfection the cells were re-transfected with the appropriate siRNA and in the case of reconstitution experiments co-transfected with either pCDNA4/TO or pCDNA4/TO FLAG 4GM. 24 h after this second transfection cells were transfected with VPg RNA isolated from MNV-infected BV-2 cells. Cells were then harvested at the appropriate times for Western blotting. Percentage reconstitution of viral translation was determined using Li-Cor Odyssey imaging whereby the levels of viral translation observed in the eIF4G siRNA- and empty vector (pCDNA)-transfected cells was set as background, and the values obtained from 4GM-expressing cells were expressed relative to the nonspecific siRNA-transfected cells. siRNA-mediated reduction of eIF4E was performed in BV-2 cells using a pool of eIF4E specific siRNAs (Santa Crux, sc-35285) transfected as described previously (31).

## RESULTS

### 

#### 

##### Proteomic Analysis of the Murine Norovirus Translation Initiation Factor Complex

**FIGURE 1.**
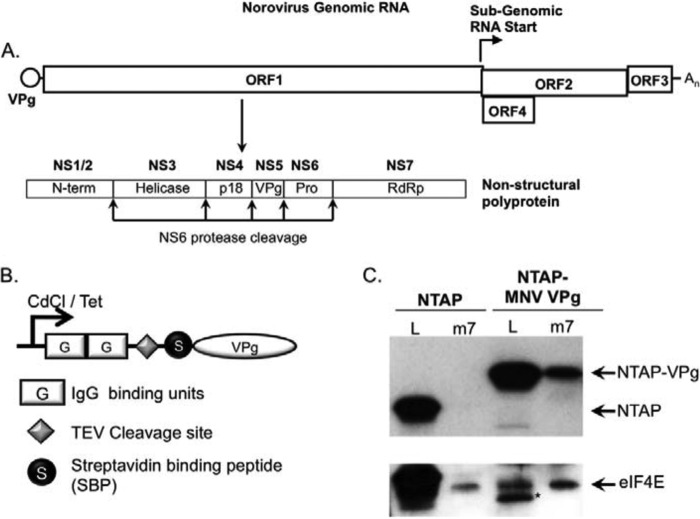
**Generation of cell lines expressing TAP-VPg.** *A*, schematic representation of the norovirus genome. The positions of the three major open reading frames (ORF1, -2, and -3) and the murine norovirus-specific open reading frame 4 (*ORF4*). The position and subgenomic RNA start site and the ORF1 polyprotein cleavage sites are highlighted. The NS1-NS7 nomenclature as proposed by Sosnovtsev (50) has been used to illustrate the identities of the mature cleavage products along with the previously described nomenclature (*N-term*, *helicase*, *RdRp* etc.). *B*, the domain layout of the TAP-tag is illustrated graphically highlighting the positions of the two protein G IgG binding domains, the tobacco etch virus (*TEV*) protease cleavage site, and the streptavidin-binding peptide along with the streptavidin binding peptide. *C*, m^7^GTP-Sepharose chromatography of cells expressing the NTAP tag or TAP-VPg fusion protein. Lysates from induced cells were incubated with m^7^GTP-Sepharose to enrich the eIF4F complex. Both lysates (*L*) and the elutions (*m7*) from the m^7^GTP-Sepharose were analyzed by Western blot for eIF4E and the presence of the protein G binding domain present in the TAP tag. An *asterisk* is used to highlight a TAP-MNV VPg degradation product present in the cell lysate.

Given the lack of functional data on the initiation factor requirements for norovirus translation initiation, we aimed first to identify the components within the VPg-initiation factor complex and then to determine which of these were functionally important. We, therefore, established a method for purifying VPg from transfected cells with any associated initiation factors. This approach is based on the well known TAP method originally described in yeast (36) but subsequently adapted for use in mammalian cells (30, 37). Murine norovirus VPg was fused at the N terminus to an affinity purification (TAP) tag consisting of two protein G domains, a tobacco etch virus protease cleavage site, and a streptavidin binding peptide (Fig. 1*B*). This expression cassette was cloned into two plasmid-based expression systems, the first an episomally maintained plasmid containing the inducible metallothionein promoter along with the Epstein-Barr virus EBNA1 protein and origin of replication (pMEP4) and the second based on pcDNA4:TO containing a CMV promoter controllable by the tetracycline repressor protein (Fig. 1*B*). Human 293T cells were used for this approach as we previously observed that, although these cells are not permissive to MNV infection due to the lack of a suitable receptor, robust MNV translation and replication occurs upon transfection of viral RNA into the cytoplasm (22). The MNV permissive cell line RAW264.7 proved to be unsuitable for this approach due to poor yields of tagged VPg recovered from these cells (data not shown).

Cell lines expressing either the tag alone (NTAP) or with TAP-VPg were generated, and expression was shown to be stimulated by the addition of cadmium chloride for the pMEP4-based system or doxycycline for the pcDNA4:TO-based expression system (data not shown). Protein expression was readily detectable by virtue of the protein G binding domains binding directly to secondary antibodies conjugated to HRP. To confirm that the TAP fusion tag did not affect the ability of VPg to associate with translation initiation factors, the ability of TAP-MNV VPg to co-purify with the eIF4F complex was determined. m7-GTP-Sepharose was used to enrich the eIF4F complex and associated factors from induced cell lines, and the levels of eIF4E or TAP-tagged protein purified in the complex determined be Western blot (Fig. 1*C*). TAP-MNV VPg was readily purified using m^7^GTP-Sepharose, whereas the TAP tag alone was not, confirming that binding to the eIF4F components was due to the VPg portion of the fusion protein.

**FIGURE 2.**
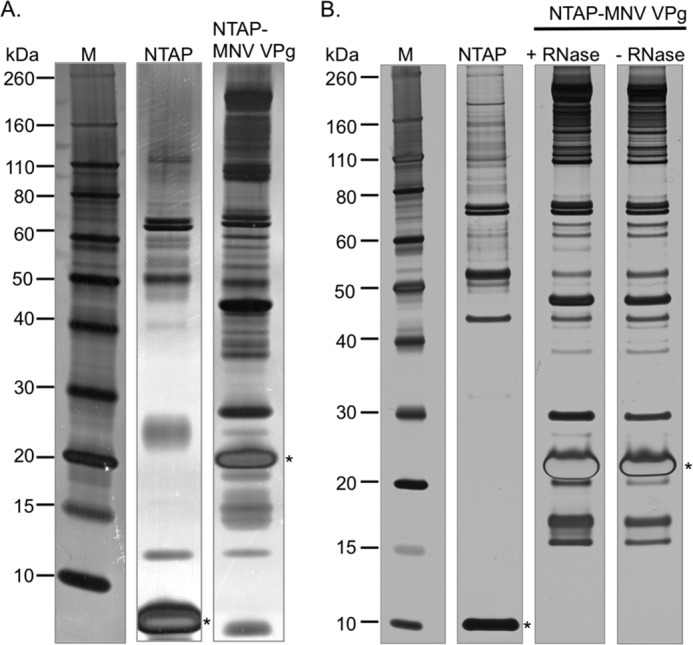
**Proteomic analysis of the norovirus translation initiation factor complex.** Final elutions after the tandem affinity purification procedure are detailed under “Experimental Procedures,” prepared from either cells expressing the NTAP tag alone or the NTAP-VPg fusion protein. *Panel A* contains the final elutions obtained from the pMEP4-based cadmium chloride inducible expression system, whereas *panel B* displays the results obtained using the pCDNA4:TO tetracycline inducible system. In the latter case, the purification was performed in the presence and absence of ribonuclease (*RNase*). An *asterisk* is used to highlight the position of the bait protein after tobacco etch virus protease digestion and biotin-mediated elution.

To identify the host factors associated with VPg, large scale affinity purifications were performed with extracts from cells expressing the TAP tag alone or TAP-MNV VPg. SDS-PAGE analysis of the purified complexes demonstrated that a number of host cell factors were co-purified with VPg that were not present in complexes isolated using the TAP tag alone (Fig. 2). The identities of the proteins isolated using the TAP method were determined by the excision from the SDS-PAGE gel, digestion with trypsin followed by mass spectrometry. Specific associations with VPg were determined by the exclusion of proteins also identified in the purification using the TAP tag alone. To eliminate the possibility of pulling down proteins due to nonspecific RNA binding ribonuclease was included in purifications performed using the doxycycline-inducible expression system as sequence analysis and our unpublished observations indicated that the norovirus VPg protein has RNA binding activity (data not shown). The inclusion of ribonuclease did not affect the profile of proteins co-purified with VPg (Fig. 2*B*).

Mass spectrometry was used to identify proteins present within the samples with a minimum of 2 unique peptides and >90% identification probability. Proteins present in the complexes isolated from cells expressing the TAP tag alone were excluded as nonspecific interacting proteins. Analysis of the proteins isolated only on VPg reproducibly identified the core eIF4F components, eIF4A, -4E, and -4G, along with PABP as factors that associate with VPg (Table 1). eIF4GI and 4GII were reproducibly identified using both experimental systems as were eIF4E, eIF4AI, and -4AII and PABP1 and -4. In addition, components of the eIF3 complex were found associated with VPg; the episomally maintained cadmium chloride-inducible system also identified the eIF3 complex (subunits A–I and K–M), whereas the tetracycline inducible system found subunits A–C, E, and K only. In addition to the canonical translation initiation factors, the episomally maintained expression system also identified a number of cellular factors including ribosomal proteins (P2, S25, S12, and S15a), LARP1, DDX9, IGF-BP1, tubulin, eEF1a, and ABCE1. As these proteins were not identified in both experimental systems and the focus of this study was to determine the role of canonical initiation factors in VPg-dependent translation, these additional host cell proteins factors were not examined in more detail. However, it is worth noting that some of these proteins have previously been identified as playing a role in the life cycles of other RNA viruses (discussed in more detail below).

**TABLE 1:**
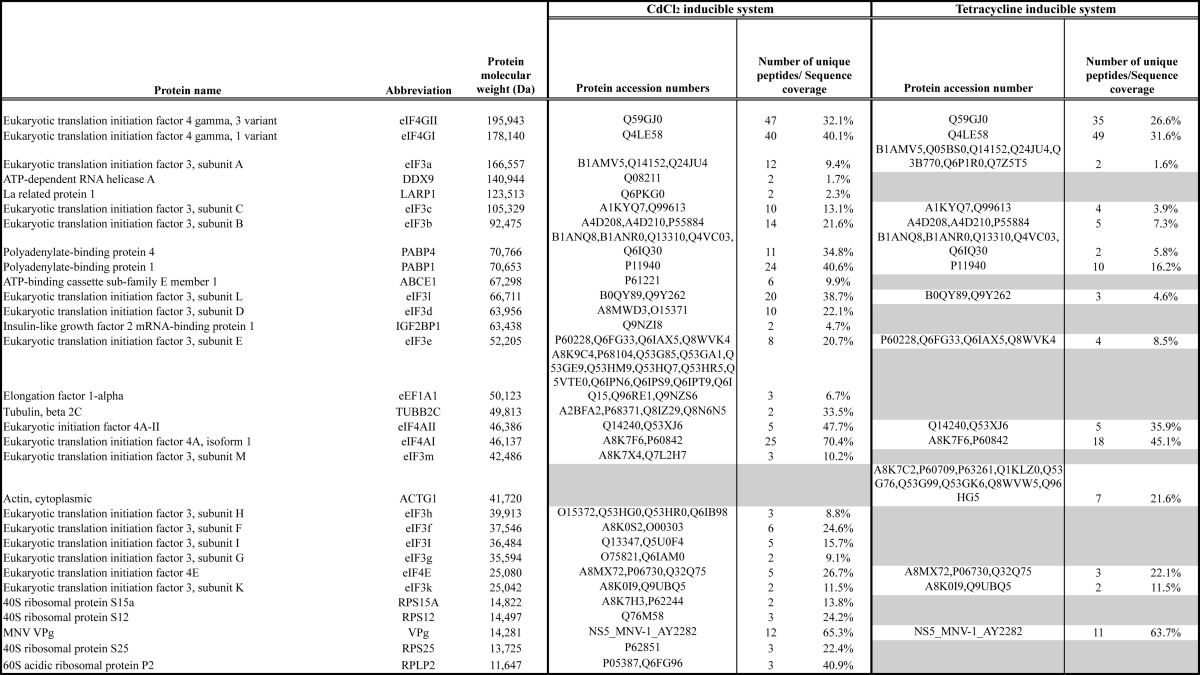
**VPg-interacting proteins identified using tandem affinity purification** VPg-containing complexes were purified from cells expressing TAP-MNV VPg expressed from either CdCl_2_ or tetracycline-inducible promoters as described under Experimental Procedures. Complexes were separated by SDS-PAGE, and the identity of proteins was determined by mass spectrometry. Proteins present in complexes purified from cells expressing the TAP tag alone were considered non-specific interacting proteins and were removed from the analysis. The identified proteins are arranged based on their predicted molecular mass (Da) with the number of unique peptides and sequence coverage for each protein also listed. Where multiple accession numbers are available for any given protein, these are also listed.

##### VPg Binds to the eIF4F Complex via an Interaction with eIF4G

**FIGURE 3.**
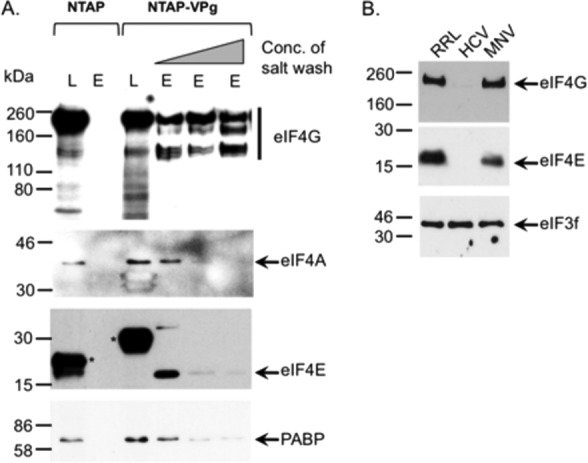
**VPg interacts with eIF4G.** *A*, cells expressing either the NTAP fusion tag or NTP-MNV VPg were used to perform tandem affinity purification as described under “Experimental Procedures”; however, before biotin elution the samples were washed with buffer containing 125 mm, 500 mm, or 1 m sodium chloride. After biotin elution, samples were concentrated, separated by SDS-PAGE, and then analyzed by Western blotting. Both lysates (*L*) and the elutions (*E*) were analyzed for the presence of eIF4G, eIF4A, eIF4A, and PABP. *Asterisks* are use to highlight the positions of the TAP tag and the TAP-VPg fusion proteins detectable in the lysate due to the presence of protein G binding domains. *B*, translation initiation complexes were purified from rabbit reticulocyte lysates (*RRL*) programmed with either the HCV IRES or VPg-linked MNV RNA by RNA-affinity purification as described under “Experimental Procedures.” Samples were subsequently analyzed by Western blot for the presence of various translation initiation factors with a sample of the input rabbit reticulocyte lysates used as a control.

To identify the interactions within the VPg-dependent translation initiation complex that are required for viral translation, we attempted to identify which components of the complex that interacted with VPg directly and contribute to viral translation. Although direct affinity measurements were not possible using the TAP purification approach, we reasoned that the interaction of VPg with the protein with the highest affinity would be resistant to increasing ionic strength. Tandem affinity purification was again performed using the murine norovirus NTAP-VPg bait protein; however, during the final washing steps increasing concentrations of sodium chloride, ranging from 125 mm to 1 m, were included to increase the stringency of the washes. Our results indicated that although the levels of eIF4E, eIF4A, and PABP isolated using NTAP-VPg were readily reduced as the ionic strength increased, the levels of eIF4G remained largely unchanged (Fig. 3*A*). These data would suggest that the norovirus VPg associates with the eIF4F complex and PABP, yet it can form a stable high affinity interaction with eIF4G. It is worth noting, however, that in addition to disrupting electrostatic interactions, increasing sodium chloride concentrations stabilize hydrophobic interactions. Therefore, these data alone do not confirm eIF4G as a direct binding partner; however, using the number of unique peptides identified for each protein (Table 1) as a semiquantitative indirect measure of protein abundance in the purified complex, it was also apparent that eIF4G was enriched in the complex with respect to the other proteins isolated.

The presence of eIF4G in the norovirus translation initiation complex was confirmed by affinity purification of assembled translation complexes from rabbit reticulocyte lysates programmed with either HCV IRES containing RNA or MNV VPg-linked RNA (Fig. 3*B*). Western blot analysis of the purified complex confirmed the presence of eIF4G and eIF4E on MNV VPg-linked RNA, both of which were absent from complexes purified on HCV IRES containing RNA as expected. In contrast, eIF3f was found associated with both MNV VPg-linked RNA and the HCV IRES containing RNA.

##### Mutations in the C Terminus of VPg Affect Initiation Factor Binding and Virus Viability

We have recently determined the structure of the MNV VPg protein (35) and demonstrated that although the central domain consists of two α helices, the N and C termini are largely disordered. To identify the regions within the norovirus VPg protein that are required for the interaction with initiation factors, we targeted several conserved amino acids for alanine mutagenesis. 14 individual point mutations in VPg were introduced into the MNV infectious clone, and protein expression was driven by coinfection of the cells with a fowlpox virus expressing T7 RNA polymerase as previously described (22). This approach was used to circumvent problems associated with reduced protein expression from non-viable viral genomes.

**FIGURE 4.**
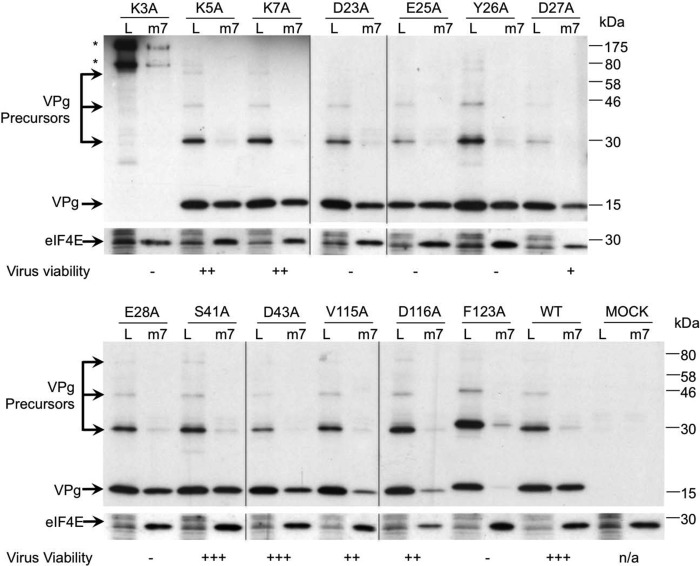
**Mutations in the C terminus of VPg affect initiation factor binding and virus viability.** BSRT7 cells infected with a fowlpox virus expressing T7 RNA polymerase were transfected with full-length cDNA clones of MNV containing either WT or various VPg mutants. 24 h post transfection cells were lysed, and the resulting lysate (*L*) was then subjected to m^7^GTP-Sepharose affinity chromatography. After purification, the proteins associated with m^7^GTP-Sepharose beads (*m7*) were separated by SDS-PAGE and analyzed by Western blotting with antisera to MNV VPg and eIF4E. *Asterisks* highlight the position of high molecular mass VPg-containing precursors formed as a result of incorrect polyprotein processing. Note that all Western blots were performed at the same time, and identical exposures were used to generate the figure shown. The m^7^GTP-Sepharose data presented is a single representative dataset from at least three independent repeats. The effect of VPg mutations on virus replication was also summarized in this figure. Virus viability is expressed as virus yield 24 h post transfection relative to wild type (+++) as assayed by >5 independent experiments. Typical yields of wild type virus were 1–5 × 10^4^ TCID50 units. −, no virus detected; +, up to 100 TCID50 detected; ++, up to 1000 TCID50; +++, up to WT levels of virus detected, typically >10,000 TCID50 per ml.

The effect of each mutation on virus viability was assayed using virus yield 24 h post transfection, and the effect of mutations on the ability of VPg to associate with initiation factors was assessed by the enrichment of the eIF4F complex using m^7^GTP-Sepharose followed by Western blotting for VPg. The mutation K3A ablated virus recovery, but this effect was mostly due to an effect on polyprotein processing as large VPg-containing precursors, not typically observed during infection, were readily detected (highlighted with an *asterisk* in Fig. 4). The mutations K5A and K7A, which reduced virus yield by 10-fold, had no appreciable effect on initiation factor binding or, as shown during our previous work, the ability of the VPg to function as a template for covalent attachment of RNA to VPg by polymerase-mediated nucleotidylation (35). Our previous work has highlighted that mutations in the region Asp-23 to Glu-28, most of which are lethal for virus viability, alter the ability of VPg to function as a template for nucleotidylation (35), but none of these was found to affect the ability of VPg to bind initiation factors. In contrast, the mutations V115A, D116A, and F123A altered the levels of infectious virus produced; a reduced virus yield was observed in the case of V115A and D116A, whereas the mutation F123A completely ablated infectious virus production. Although the majority of the mutants had little or no reproducible effect on the ability of VPg to associate with eIF4F compared with wild type VPg, the mutants V115A, D116A, and F123A showed a consistently reduced interaction with eIF4F (Fig. 4). The mutation F123A clearly resulted in the greater reduction in the amount of VPg recovered using m^7^GTP-Sepharose; low levels of VPg F123A were reproducibly detected only upon long exposure of the Western blots (data not shown). The degree to which the mutation affected virus recovery appeared to correlate with the ability of VPg to be co-purified with initiation factors (Fig. 4). In addition, sequence analysis indicated that the V115A mutant was unstable and readily reverted back to WT after repeated passage in cell culture (data not shown). These data would indicate that the disordered C terminus of the norovirus VPg protein contains amino acids involved in the interaction with eIF4G.

**FIGURE 5.**
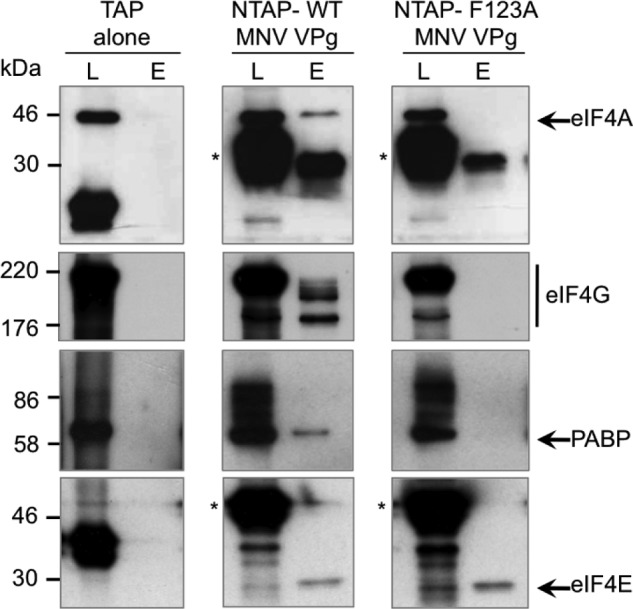
**The F123A mutation abolishes the VPg-eIF4G interaction.** Tandem affinity purification was performed on human 293T cells transiently transfected with plasmids containing the TAP tag alone, TAP-wild type MNV VPg (WT), or TAP-MNV VPg containing the mutation F123A. Samples of the cell lysate (*L*) or the eluted purified complex (*E*) were analyzed by Western blot for eIF4A, eIF4G, PABP, and eIF4E. *Asterisks* are used to highlight the position of the TAP-VPg fusion proteins detected by the binding of the primary antibody to the protein G domains present in the TAP tag.

To further validate our observation that the C terminus of VPg contributes to the interaction with eIF4G, we performed small scale tandem affinity purification with both the WT and the F123A mutant form of MNV VPg and examined the purified complex for eIF4E, eIF4A, eIF4G, and PABP. The mutation F123A ablated the ability of VPg to co-purify eIF4G, eIF4A, and PABP but did not affect the ability of NTAP-MNV VPg to co-purify eIF4E (Fig. 5), suggesting that the eIF4E and eIF4G binding regions on VPg are distinct. The ability of the F123A VPg mutant to interact with eIF4E is in agreement with the low levels of this mutant isolated using m^7^GTP-Sepharose (Fig. 4) and the ability of recombinant F123A VPg to bind eIF4E using a capture ELISA assay (data not shown). These data would suggest that the direct interaction of VPg with eIF4G largely determines the eIF4F binding capacity of the norovirus VPg protein.

##### VPg Interacts with the Central Domain of eIF4G via a Direct Protein-Protein Interaction

**FIGURE 6.**
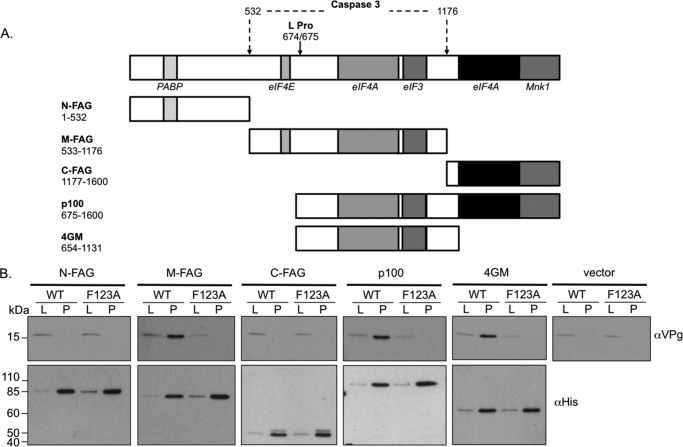
**VPg interacts with the central domain of eIF4G.** *A*, cleavage map of eIF4GI highlighting the positions for translation factors. The *arrow* and the *corresponding numbers* illustrate the locations of the individual protease cleavage sites mediated by the cellular proteins caspase 3 or the viral protease Lpro. The expression constructs used in the assay are also illustrated along with the specific amino acids residues encompassed within the construct. *B*, nickel-affinity purification of His-eIF4G fragments. Cells previously infected with a foxpox virus expressing T7 RNA polymerase were co-transfected with the various eIF4GI expression constructs (or empty vector) and either a wild type murine norovirus cDNA clone (*WT*) or one containing the F123A mutation in VPg (*F123A*). Lysates (*L*) or the purified complex (*P*) were prepared and used for nickel-affinity purification followed by Western blotting for the presence of wither VPg or the recombinant protein (His).

To determine which portions of eIF4G interact with the MNV VPg protein, we examined the ability of various previously described domains of eIF4GI to co-purify VPg when expressed in mammalian cells. Based on our previous observation that foot and mouth disease virus L protease-mediated cleavage of eIF4G had no effect on norovirus translation *in vitro* (25), we hypothesized that the interacting domain would be within the p100 region of eIF4G (amino acids 675–1600). Mammalian cells, previously infected with a recombinant fowlpox virus expressing T7 RNA polymerase, were transfected with plasmids expressing His-tagged derivatives of the various eIF4GI domains (Fig. 6*A*) and cDNA clones containing either a WT MNV genome or one containing the F123A mutation in VPg. The ability of the eIF4G domains to interact with VPg was then determined by Western blot analysis of the complex isolated using nickel-agarose to affinity purify the His-eIF4GI domains. This approach was used in place of authentic infection to enable the use of the F123A VPg mutant as a specificity control as this cDNA clone does not produce infectious virus (Fig. 4). A reproducible interaction was found between WT MNV VPg and the fragments of eIF4GI expressed from the constructs M-FAG, p100, and 4GM (Fig. 6*B*). In contrast, neither of the N or C-terminal fragments, N-FAG or C-FAG, was found to interact with VPg. Specificity of the interaction was also confirmed using the mutant F123A, which abolished the interactions observed between wild type VPg and the eIF4G fragments. These data indicate that the VPg binding site lies within residues 654–1131 of eIF4GI, a region known to contain both eIF4A and eIF3 binding sites (7).

**FIGURE 7.**
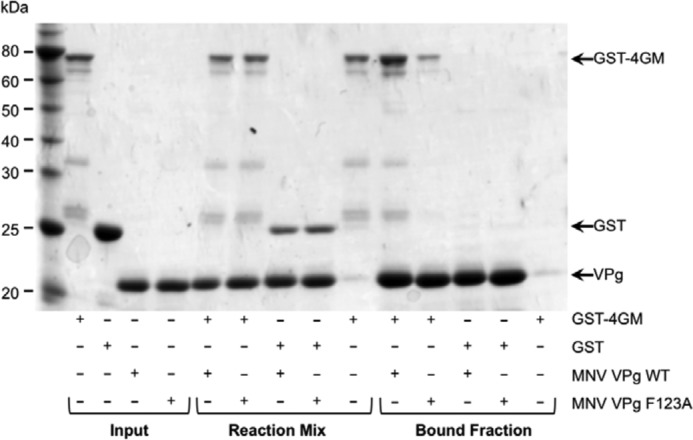
**VPg binds the central domain of eIF4G via a direct protein-protein interaction.** The ability of recombinant eIF4G central domain (*4GM*) to interact with either wild type MNV VPg (WT) or the F123A MNV VPg mutant was examined using a His-tag pulldown assay. Recombinant GST or GST-4GM was mixed with purified His-tagged WT or F123A MNV VPg. and the resulting complex was purified using cobalt affinity chromatography. Samples of the purified proteins (*Input*), the mixtures of the proteins before purification (*Reaction mix*), and the final bound fraction were separated by SDS-PAGE and analyzed by Coomassie Blue staining.

To confirm that the VPg-eIF4GI interaction was not mediated by a cellular partner or nucleic acid co-purified with the eIF4G fragments from the mammalian cells, *E. coli*-expressed recombinant protein was used to perform a similar assay with recombinant norovirus VPg. The ability of GST-tagged 4GM fragment or GST alone to bind to recombinant His-tagged WT or F123A MNV VPg was examined using a His-tag pulldown assay (Fig. 7). Although GST-4GM readily co-purified with WT MNV VPg, GST did not. Moreover, the F123A mutation in the MNV VPg protein significantly reduced the interaction of VPg with eIF4G in this assay (Fig. 7). These data confirm that the VPg-eIF4G interaction occurs via a direct protein-protein interaction and does not require additional cellular proteins.

##### Neither the eIF4E-eIF4G Interaction nor High Levels of eIF4E Are Required for Norovirus Translation and Replication

**FIGURE 8.**
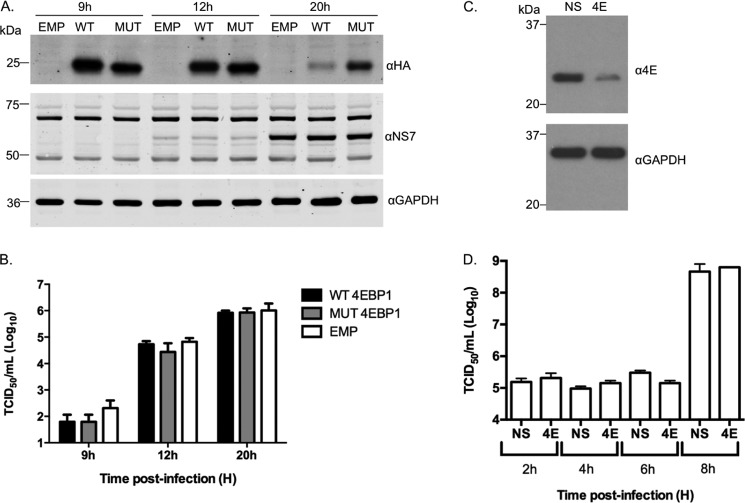
**Reduced levels of eIF4E and inhibition of the eIF4E-4G interaction are not required for efficient norovirus replication.** *A*, MNV permissive microglial cells BV2 were transiently transfected with either an empty plasmid vector (*EMP*) or plasmids expressing HA-tagged derivatives of wild type (*WT*) or a non-phosphorylatable mutant of 4E-BP1 (*MUT*). Cells were then infected with MNV1 at a multiplicity of infection of 3 TCID_50_/cell, and viral protein expression as well as virus titer (*B*) was determined at various times post infection. *C*, BV2 cells transfected with either non-targeting siRNA or an siRNA directed against eIF4E were infected with MNV1 at a multiplicity of infection of 10 TCID_50_/cell, and the effect on viral titer examined at various times post infection (*D*).

We previously reported that the norovirus VPg protein can interact with eIF4E directly but have also shown that eIF4E is dispensable for norovirus translation *in vitro* (25). To explore whether the eIF4E-eIF4G interaction is required for norovirus translation during an authentic viral life cycle, we examined the effect of overexpression of 4E-BP1 on virus translation and replication in permissive cells. 4E-BP1 interferes with the eIF4E-eIF4G interaction, and 4E-BP1 overexpression results in the inhibition of eIF4G recruitment to the 5′ end of mRNA. This process is regulated by phosphorylation of 4E-BP1 as only the phosphorylated form of 4E-BP1 interacts with eIF4E (38). Wild type or a non-phosphorylatable mutant version of 4E-BP1 were overexpressed in cells, and the effect on MNV replication was examined (Fig. 8, *A* and *B*). The overexpression of 4E-BP1 resulted in inhibition of the eIF4E-4G interaction and had no effect on MNV viral protein expression (Fig. 8*A*), virus titer (Fig. 8*B*), or viral RNA levels (not shown), whereas the ability of eIF4G to be co-purified with eIF4E on m^7^GTP-Sepharose was significantly reduced by ∼80 and ∼60% for the WT and mutant forms of 4E-BP1, respectively (data not shown). Further evidence that eIF4E does not play a direct role in norovirus translation was obtained by examining the effect of siRNA-mediated reduction of eIF4E on norovirus replication (Fig. 8*C*). Reduction of cellular eIF4E levels by >90% (Fig. 8*C*) had no appreciable effect on MNV virus titer under the conditions used (Fig. 8*D*), suggesting that high levels of eIF4E are not required for efficient virus replication in immortalized microglial cells.

##### The eIF4G Central Domain Is Required for Norovirus VPg-dependent Translation

**FIGURE 9.**
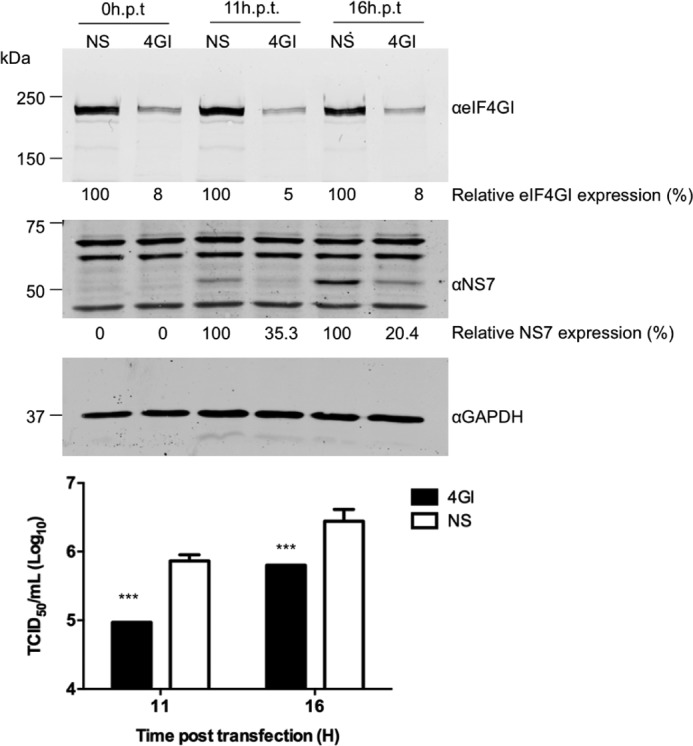
**eIF4G is required for efficient norovirus translation and replication.** HEK 293T cells were transfected with specific eIF4GI siRNAs or a nonspecific (*NS*) siRNA. The cells were re-transfected with the siRNA at 24 hours post-transfection (h.p.t.) to improve the knockdown efficiency. Thereafter, the cells were transfected with MNV VPg-linked RNA. Western blots were performed on lysates harvested at 11 and 16 hours post transfection of RNA to examine the efficiency of eIF4GI silencing and the translation of NS7 and GAPDH. The *numbers below the figures* represent the percentage of protein expression relative to the control, which were quantified by densitometry using ImageJ. The yield of virus was determined in RAW 264.7 cells from two independent experiments, expressed as TCID_50_/ml and compared with NS control. Transfections were performed in triplicate, and the *error bar* indicates the S.E. Statistical significance was determined by one-way analysis of variance and is represented by the *p* values. *p* < 0.001 (***).

**FIGURE 10.**
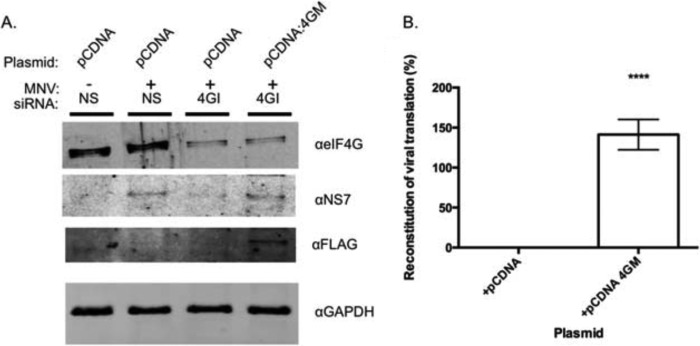
**The central domain of eIF4G is sufficient for norovirus translation.** 293T cells transfected with siRNA directed against eIF4G (*4GI*) or non-specific siRNA (*NS*) were co-transfected with plasmids encoding either empty vector (*pCDNA*) or the minimal VPg binding domain from eIF4G (*pCDNA 4GM*). *A*, Western blots showing successful knockdown of 4G and reconstitution of viral protein expression upon co-transfection with FLAG-tagged 4GM. *B*, the ability of 4GM to reconstitute viral protein expression in eIF4G depleted cells is represented as % reconstitution, relative to a nonspecific siRNA-treated control as described under “Experimental Procedures.” Quantification was performed on a Li-Cor Odyssey imager on data obtained from quadruplicate independent biological samples. *Error bars* represent S.E. with significance determined by one-way analysis of variance (****, *p* = <0.0001).

To determine if eIF4G plays a role in the translation of the VPg-linked norovirus RNA, we examined the effect of eIF4G siRNAs on norovirus replication (Fig. 9). Due to difficulties in reproducibly reducing eIF4G levels in mouse macrophage or microglial cells permissive to MNV infection, we used human cells instead. Transfection with eIF4G siRNAs was followed by transfection with viral VPg-linked RNA. This approach was based on the observation that MNV VPg-linked RNA is infectious when transfected in human cells (22), which suggest that all the cellular machinery required for efficient viral RNA translation and replication is conserved. At 11 and 16 h post transfection samples were analyzed for viral protein by Western blot and the production of infectious virus (Fig. 9). A significant reduction in both viral protein expression and virus replication was observed at both time points; the virus yield was reduced 8.9-fold (∼89%) after 11 h and 5.6-fold (∼82%) after 16 h. To confirm that the observed effect on viral translation was specific and that the central domain of eIF4G was sufficient for viral translation, we examined the ability of the 4GM domain alone to complement eIF4G siRNA-treated cells. Fluorescence-based Western blotting and quantification of multiple independent experiments confirmed that the expression of the 4GM domain of eIF4G was sufficient to restore viral translation in eIF4G siRNA-transfected cells (Fig. 10).

## DISCUSSION

Until recently the novel mechanism of calicivirus genome translation has been poorly characterized, largely due to the inability to culture many members of the virus family in immortalized cells. FCV, a member of the *Vesivirus* genus, has until very recently provided the only tractable experimental system with which to characterize this novel mechanism of calicivirus VPg-primed viral genome translation. However, with the discovery of a cultivatable member of the *Norovirus* genus, namely MNV, it has now been possible to use genetic and biochemical assays to examine the role of protein-protein interactions in the norovirus life cycle. Our previous work on norovirus translation using MNV as a model system demonstrated a direct interaction with eIF4E, which at least *in vitro* was not required for viral translation, but also implicated a functional role for the helicase eIF4A in viral translation initiation (25, 26). In agreement with other earlier observations from our laboratory (25), we have now used a proteomics approach to observe that the MNV VPg protein can be used to affinity purify the entire eIF4F complex from cells, including eIF4G. In addition to eIF4F, we identified PABP, components of the eIF3 complex, and a number of cellular proteins previously implicated in protein synthesis as associates of VPg (Table 1). These additional proteins include LARP1, ABCE1, DDX9, IGFBP1, eEF1A1, and various ribosomal proteins. The association of VPg with these proteins is likely to be indirect and via their interaction with other components of the initiation factor complex. Although the role of these proteins in norovirus translation was not examined here, we recently identified eEF1A as a component of the ribonucleoprotein complex found associated with conserved RNA structures at the 5′ extremity of the MNV genome (31). It is, therefore, possible that subsequent to recruitment of the complex to the 5′ end of the norovirus genome via VPg, additional RNA-protein interactions occur that stabilize the complex and contribute to norovirus translation.

In addition to the eIF4F complex, we also identified eIF3 components present in the VPg-containing complex isolated from cells. This is in agreement with previous reports suggesting norovirus VPg may interact directly with eIF3 (28). The eIF3 binding site on eIF4G has been mapped to a short ∼90-amino acid stretch (39, 40), and reports suggest that it is the eIF3 subunits -c, -d, and-e that interact with eIF4G (41, 42). As expected all three of these subunits were identified as a component of the complex by mass spectrometry. It, therefore, appears that VPg is capable of making multiple contacts with components of the pre-initiation complex, some of which function directly in translation initiation, whereas others may contribute to the regulation of host cell translation by virus infection. Both eIF4GI and eIF4GII were readily detected in VPg-containing complexes purified from cells. Our functional work to date has focused on the role of eIF4GI on norovirus translation, but the role of other eIF4G isoforms on norovirus translation remains to be determined, particularly given that previous studies have indicated some evidence of functional redundancy (43). The inability of the TAP-VPgF123A to co-purify eIF4E with eIF4G (Fig. 5) would suggest that either the mutation blocks the eIF4E-eIF4G association or that VPg interacts with eIF4E and eIF4G independently. Our previous data and that presented in the current study would suggest that VPg is likely to make multiple contacts with the eIF4F complex, including interactions with eIF4E and eIF4G. The location of the eIF4E-binding site on VPg has yet to be determined but will be facilitated with the availability of the F123A mutant.

We recently analyzed the solution structure of the core domain of VPg using nuclear magnetic resonance (NMR) spectroscopy and found that the MNV VPg protein consists of a compact structured core formed by a pair of α-helices that is flanked by long, flexible N and C termini (35). The region we have identified as being involved in the direct interaction with eIF4G, namely the C-terminal domain, is disordered and, therefore, is likely to adopt a fixed structure only upon interaction with eIF4G. The minimal eIF4G binding domain has yet to be fully determined, but as the C-terminal 17 amino acids of human Norwalk virus and MNV VPg proteins are highly conserved (15 of 17 amino acids are 100% identical), it is likely that the interaction with eIF4G is a feature conserved between murine and human noroviruses. Further studies on the role of this interaction in the human norovirus life cycle are under way.

Although we have been able to demonstrate a functionally relevant interaction between the norovirus VPg and eIF4G, the role of the VPg-eIF4E interaction remains unclear. Our initial experiment on the identification of a norovirus VPg-eIF4E interaction weas performed before the widespread availability of a cultivatable norovirus with which to probe the role of the interaction. Our subsequent work, however, demonstrated that although a VPg-eIF4E interaction is essential for translation of FCV RNA, a member of the *Vesivirus* genus, this interaction is not required for MNV translation (25), a *Norovirus*. Fitting with these observations, we observe here that reducing eIF4E levels or reducing the interaction between eIF4E and eIF4G in cells appears not to affect virus replication under the experimental conditions used (Fig. 8). It is important to note that in both these approaches low levels of eIF4E remained (Fig. 8*C*) and 4E-BP1 expression in the MNV permissive cells did not completely block the eIF4E-4G interaction. Therefore, further studies on the role of eIF4E during the norovirus life cycle are clearly warranted, but it is worth noting that in addition to a direct role in cap binding for translation initiation, eIF4E plays numerous roles in the regulation of gene expression. For example the interaction of eIF4E with eIF4G relieves an autoinhibitory function of eIF4G on the RNA helicase eIF4A (44). Therefore, recruitment of eIF4E to the 5′ end of norovirus RNA would be predicted to enhance the helicase activity of the eIF4A that has formed a complex with the VPg-associated eIF4G. The interaction of VPg with eIF4E may also play a role in regulating eIF4E phosphorylation, as eIF4E is phosphorylated by MNK1 via an interaction with the eIF4G scaffold protein (45). Phosphorylated forms of eIF4E also play a role in the translation of a subset of cellular mRNAs, including those involved in the response to viral infection (46). Our ongoing studies have suggested that the VPg-eIF4E interaction may simply function to recruit eIF4E to facilitate the formation of polysome-associated phosphorylated eIF4E.^4^ The role of the VPg-eIF4E interaction is, therefore, most likely involved in controlling the host response to infection, but further studies are required.

eIF4G is also known to be phosphorylated (47), although the physiological relevance of eIF4G phosphorylation has become apparent only recently; data have highlighted that phosphorylation of eIF4G by protein kinase Cα (PKCa) increases an interaction with MNK1 (48). Independent observations indicate that eIF4G phosphorylation is stimulated during norovirus replication in cell culture and that this phosphorylated form of eIF4G is associated with MNV VPg in infected cells (29). The impact and functional relevance of initiation factor phosphorylation on the norovirus life cycle is currently under study.

The novel mechanism of VPg-primed translation initiation is not unique to caliciviruses and has been widely characterized in a number of plant viruses where it contributes to susceptibility to infection (for review, see Ref. 27). In the case of the potyvirus tobacco etch virus, VPg interacts with both eIF4E and eIFiso4E, increasing the affinity of the eIF4F complex for tobacco etch virus RNA (49). The effect of the norovirus VPg-eIF4G interaction on the affinity of the complex for the norovirus RNA genome is as yet unknown, but it is also possible that similar allosteric effects of the VPg-eIF4G interaction on other components of the translation initiation factor complex contribute to the stabilization of the initiation factor complex and promote ribosome recruitment.

Overall our work has provided additional insight into the novel mechanism of VPg-dependent viral translation initiation. Given the key role of translation initiation in the life cycle of noroviruses, we would propose that the interaction of VPg with eIF4G might provide a suitable therapeutic target for these pathogens.

## Footnotes

4 E. Royall, A. Abdul-Wahab, N. Doyle, S. J. Morley, I. Goodfellow, L. O. Roberts, and N. Locker, manuscript in preparation.

## Footnotes

3 The abbreviations used are:

eIF: eukaryotic initiation factor
MNV: murine norovirus
FCV: feline calicivirus
TAP: tandem affinity purification
hpt: hours post-transfection
HCV: hepatitis C virus
PABP: poly A binding proteins
IRES: internal ribosome entry site
GMP PNP: guanosine 5′-{β,γ-imido}triphosphate.
